# Native microalgal-bacterial consortia from the Ecuadorian Amazon region: an alternative to domestic wastewater treatment

**DOI:** 10.3389/fbioe.2024.1338547

**Published:** 2024-02-26

**Authors:** Amanda M. López-Patiño, Ana Cárdenas-Orrego, Andrés F. Torres, Danny Navarrete, Pascale Champagne, Valeria Ochoa-Herrera

**Affiliations:** ^1^ Colegio de Ciencias e Ingeniería, Universidad San Francisco de Quito USFQ, Quito, Ecuador; ^2^ Instituto de Microbiología, Universidad San Francisco de Quito USFQ, Quito, Ecuador; ^3^ Colegio de Ciencias Biológicas y Ambientales, Universidad San Francisco de Quito USFQ, Quito, Ecuador; ^4^ Department of Civil Engineering, Queen’s University, Kingston, ON, Canada; ^5^ Department of Environmental Sciences and Engineering, Gillings School of Global Public Health, University of North Carolina at Chapel Hill, Chapel Hill, NC, United States; ^6^ Escuela de Ingeniería, Ciencia y Tecnología, Universidad del Rosario, Bogotá, Colombia

**Keywords:** microalgal-bacterial consortia, wastewater treatment, nutrients removal, organic matter removal, removal rates

## Abstract

In low-middle income countries (LMIC), wastewater treatment using native microalgal-bacterial consortia has emerged as a cost-effective and technologically-accessible remediation strategy. This study evaluated the effectiveness of six microalgal-bacterial consortia (MBC) from the Ecuadorian Amazon in removing organic matter and nutrients from non-sterilized domestic wastewater (NSWW) and sterilized domestic wastewater (SWW) samples. Microalgal-bacterial consortia growth, in NSWW was, on average, six times higher than in SWW. Removal rates (RR) for NH_4_^+^- N and PO_4_^3−^-P were also higher in NSWW, averaging 8.04 ± 1.07 and 6.27 ± 0.66 mg L^−1^ d^−1^, respectively. However, the RR for NO_3_^−^ -N did not significantly differ between SWW and NSWW, and the RR for soluble COD slightly decreased under non-sterilized conditions (NSWW). Our results also show that NSWW and SWW samples were statistically different with respect to their nutrient concentration (NH_4_^+^-N and PO_4_^3−^-P), organic matter content (total and soluble COD and BOD_5_), and physical-chemical parameters (pH, T, and EC). The enhanced growth performance of MBC in NSWW can be plausibly attributed to differences in nutrient and organic matter composition between NSWW and SWW. Additionally, a potential synergy between the autochthonous consortia present in NSWW and the native microalgal-bacterial consortia may contribute to this efficiency, contrasting with SWW where no active autochthonous consortia were observed. Finally, we also show that MBC from different localities exhibit clear differences in their ability to remove organic matter and nutrients from NSWW and SWW. Future research should focus on elucidating the taxonomic and functional profiles of microbial communities within the consortia, paving the way for a more comprehensive understanding of their potential applications in sustainable wastewater management.

## 1 Introduction

According to the United Nations. (UN), over 80% of global wastewater (WW) is discharged into the environment without undergoing any treatment (UN, 2017). This practice exposes nearly 22% of the world’s population to water potentially contaminated with fecal matter, leading to the spread of diseases such as typhoid, polio, hepatitis, cholera, and dysentery (UN, 2017). In Ecuador, a low-middle-income country (LMIC), the situation is particularly challenging and complex (Borja-Serrano et al., 2020). Domestic and industrial WW effluents are released through the public sewer system directly to freshwater bodies without any treatment (Leon et al., 2020). The latter serve as primary sources of fresh water in rural areas for domestic activities such as personal hygiene, laundry, food preparation, and drinking (SENAGUA, 2016; Vinueza et al., 2021). Consequently, there is a pressing need to shift the paradigm of wastewater management from one that is disposable to a model that is treatable, reusable, recyclable, and geared toward resource recovery (Khan et al., 2018).

Under Sustainable Development Goal 6, the UN proposed to reduce the proportion of untreated wastewater and increase safe reuse by 2030 (Gonçalves et al., 2017; UN, 2018). Several wastewater treatment strategies exist today, including coagulation-flocculation, electrochemical treatment, adsorption, ion-exchange, ultrafiltration, chemical and physical precipitation, and activated sludge (AS) wastewater treatment (Najafi et al., 2022; Guo et al., 2023). These conventional treatment methods require intensive energy inputs and extensive land, and incur high operational and maintenance costs (Luo et al., 2019).

As an alternative, microalgal-bacterial consortia (MBC) have emerged as one of the passive wastewater treatment systems exhibiting advantages in terms of energy demand, environment performance, ease of implementation, and costs (Chan et al., 2022). The microalgal–bacterial interaction reveals the mutual relationship of microorganisms where algae are primary producers of organic compounds from CO_2_, and heterotrophic bacteria are secondary consumers decomposing the organic compounds produced from algae (Khoo et al., 2021). Microalgae-based WW remediation is a sound alternative for removing organic matter and nutrients (C, N, and P) from WW, due to the low energy consumption of aeration, strong nutrient removal capacity, low greenhouse gas emissions, and high resource recovery potential. The algal biomass produced during the remediation process can also be used to recover valuable bioproducts (fertilizers, biofuels, and pharmaceuticals) to improve the economics of the WW treatment process (Guo et al., 2023).

In 2015, Benítez *et al.* found that native Ecuadorian *Chlorella sp*. alone could successfully treat synthetic wastewater (Benítez et al., 2019). Currently, this field has become more ambitious in further enhancing bioremediation with microalgal-bacterial consortia (MBC).

Nutrient removal efficiencies in wastewater dropped from ammonium (NH_4_^+^-N) (100%), nitrate (NO_3_^−^-N) (15%) and phosphate (PO_4_^3-^-P) (36%) to 75%, 6% and 19%, respectively, when switching from using microalgal-bacterial consortia (MBC) to microalgae alone (Velázquez and Rodríguez-Barrueco, 2007). Similarly, Liang *et al.*, reported that 78% of NH_4_^+^-N can be removed in a microalgal-bacterial system, while 29% in a single microalgae system and purely 1% in a single bacteria system (Liang et al., 2013). Although several studies have proven the maximization of wastewater treatment efficiency obtained by using these symbiotic self-sustaining systems, there is a latent biological gap that needs to be filled between the complex interactions among microalgal-bacterial consortia (MBC) and the role of the native wastewater microbial community; which entails, a lot more than just nutrient exchange (Mohsenpour et al., 2021). The dynamics of microbial communities change depending on the environment, going from mutualistic under nutrient-replete to competitive under nutrient-deplete conditions (Mayali, 2018). Microorganisms either cooperate through the exchange of extracellular metabolites, vitamins, and siderophores to increase nutrient uptake rates and biomass productivities, or compete by metabolite excretion to produce bactericidal or micro-algicidal effects (Cydzik-Kwiatkowska and Zielińska, 2016). Consistently, the research pathway in this scientific field has been directed towards the use of two different medium cultures: non-sterilized wastewater (NSWW) and sterilized wastewater (SWW) (Unc et al., 2017). The few studies that exist on this topic have shown that MBC nutrient removal efficiencies vary significantly depending on which medium they grow on; as NSWW contains natural micro-fauna that does not exist in SWW. For instance, Lv *et al.*, found that wastewater (non-sterilized circumstances) enhances nutrient removal and lipids accumulation (Lv et al., 2017).

The development of robust, resilient, and low-cost wastewater treatment systems that are easy to operate and maintain for the degradation of organic matter and the removal of nutrients [nitrogen (N) and phosphorous (P)], are common challenges in the global south (Gallego-Schmid and Tarpani, 2019). Therefore, the utilization of native MBC bioremediation seems to be an efficient wastewater treatment alternative in LMIC such as Ecuador. Hence, the objective of this study is to evaluate and compare the capability of six native microalgal-bacterial consortia (MBC) native to the Ecuadorian Amazon to remove organic matter and nutrients from NSWW and SWW samples. Removal efficiencies as well as removal rates in non-sterilized and sterilized domestic wastewater samples were also calculated to evaluate the performance of the different MBC employed in this study. Taking this path will lead us one step further towards generating knowledge in native microalgal-bacterial consortia wastewater treatment efficiency, their behavior under the absence/presence of the autochthonous microbial community present in wastewater samples, and the influence of the composition of wastewater during the bioremediation process.

## 2 Materials and methods

### 2.1 Wastewater collection and characterization

#### 2.1.1 Wastewater sampling

The collection of wastewater samples was performed following the methodology described by the Standard Methods for the Examination of Water and Wastewater section 1060 B (APHA, 2002). Flow values were measured in advance every 30 min to obtain a plot of volumetric flow as a function of time. This information was utilized to establish the relative proportions of wastewater that was collected every 30 min for 8 h to reach a total volume of 1 L. Then, three composite samples of domestic wastewater were collected from the discharge point at Universidad San Francisco de Quito (Quito, Ecuador) from October 2018 to January 2019. Composite samples were generated by collecting individual samples every 30 min, for 8 h, in volumes proportional to the flow of the discharge point. Samples were cumulatively collected in 1 L amber glass bottles that were transported to and stored in cool conditions at the Laboratory of Environmental Engineering at Universidad San Francisco de Quito (LIA-USFQ). In addition to volumetric flow, physical parameters like dissolved oxygen (DO) and temperature (T) (SM 4500-O A), pH (SM 4500 H^+^ B), and electrical conductivity (EC) (SM 2510) were measured *in situ* using a Thermo Scientific Orion 5-Star portable multi-parameter (Thermo Specific Electrode, Orion).

#### 2.1.2 Analytical methods

Analytical methods described in Standard Methods (APHA, 2002) were used in this study as follows: Ammonium (NH_4_^+^-N) (SM 4500-NH_3_-D), Chloride (Cl^−^) (SM 4500 Cl-D) and fluoride (F^−^) (SM 4500 F-C) were tested potentiometrically using a Thermo Scientific Orion 5-Star portable multi-parameter and its corresponding probes (Thermo Specific Ion Selective Electrode, ISE Orion). Nitrate (NO_3_^−^-N) (SM 4500-NO_3_^-^-B), Phosphate (PO_4_^3-^-P) (SM 4500-P-B), Chemical oxygen demand (COD) (Total and Soluble COD) (SM 5520-B) and Sulphide (S^2-^) (SM 4500 S2-D) were measured using a visible spectrophotometer (Thermo Scientific Inc. GENESYS 30. United States). Likewise, Total Kjeldahl Nitrogen (TKN) (ISO 5663-1984, EPA 351.3 and AOAC 973.48) was measured colorimetrically using a SpeedDigester K-436 and Kjeldahl K-360 sampling system. Sulphate (SO_4_^2-^) (SM 426-C) and Total suspended solids (TSS) (SM 2540-D) were tested gravimetrically using glass microfiber filters (Whatman Grade 934-AH). Biochemical oxygen demand (BOD_5_) (SM 5219 B) was measured in a 5-day period using the OxyTop system.

### 2.2 Native microalgal-bacterial consortia cell propagation and adaptation

The native microalgal-bacterial consortia (MCB) were collected from different locations within Lago Agrio, Ecuadorian Amazon Region. The geographical coordinates for all consortia are M1 (0.12714, −76.84980), M2 (0.11993, −76.85534), M3 (0.1231875, −76.8738837), M4 (0.10895, −76.87162;), M5 (0.10909, −76.87186) and M6 (0.10943, −76.87168). Supplementary Figure S1 presents geographical locations for all MBC evaluated in this study.

The collected consortia samples were filtered through cellulose filter papers (Whatman Grade 1) to remove coarse materials (rocks and vegetation). For each MBC, 2.5 mL of the filtered sample was added to 47.5 mL of either non-sterilized wastewater (NSWW) or sterilized wastewater (SWW), to give a total volume of 50 mL. The inoculated solution was incubated in a photobioreactor (PBRs) agitated at 100 rpm in an orbital shaker (ACTUM HD-4000) to stimulate cellular growth. Fifteen days later, the cell adaptation in PBRs aerated with an air pump using atmospheric air flux of 1 L s^−1^ mixed through a diffuser began. In the second phase, 50 mL cell suspensions from the previous phase were inoculated with 450 mL of NSWW or SWW to give a total volume of 500 mL PBRs. In both phases, 3 replicates of every PBR in NSWW or SWW were illuminated with artificial light by tubular fluorescent lamps 20 W OSRAM and LED lights during 12-h photoperiods (1000 lx) at room temperature (24°C ± 0.5°C). After the second phase of propagation, cellular densities for the microalgal-bacterial consortia ranged from 1.6 to 2.0 g L^−1^; sufficient to conduct organic and nutrient removal bioassays.

### 2.3 Batch bioassays

#### 2.3.1 Composition, set up and operational conditions

Two different batch bioassay compositions were used in this study: (1) treatment (Ts) and (2) abiotic control (AC). To account for biotic nutrient removal, Ts containing 950 mL of NSWW or SWW and 50 mL of non-sterilized native microalgal-bacterial consortium were used. To account for abiotic nutrient removal, AC containing 950 mL of NSWW or SWW (absence of native microalgal-bacterial consortium) and 50 mL of distilled water were placed. Therefore T1, T2, T3, T4, T5, and T6 correspond to experimental assays inoculated with MBC M1, M2, M3, M4, M5, and M6, respectively, at a concentration of 5% v/v of native microalgal-bacterial consortium to have an initial cell density of 100 mg L^−1^, dry weight as described in (Benítez et al., 2019).

All batch bioassays containing NSWW or SWW were performed in triplicate and set up in 1000 mL PBRs aerated continuously with an air pump using an atmospheric air flux of 1 L s^-1^ mixed through a diffuser at room temperature (24°C ± 0.5°C) and pH of 7.0 ± 0.5. The PBRs were illuminated with artificial light by tubular fluorescent lamps 20 W OSRAM and LED lights during 12-h photoperiods (1000 lx) for 15 days. Furthermore, physical-chemical parameters such as DO, T, pH, and EC were measured every 3 days to monitor native MBC operational conditions.

#### 2.3.2 Performance conditions evaluation

##### 2.3.2.1 Biomass concentration

Biomass concentration was quantified in each bioassay every 3 days to evaluate the growth performance of native MBC in NSWW and SWW. Measurements were taken using a gravimetric method after filtration of 20 mL of the sample with a pre-dried glass microfiber filter (Whatman Puradisc pore size: 0.45 µm and diameter: 47 mm) and drying both together, the filter and biomass, at 105°C in a moisture analyzer (COBOS) until constant weight.

The biomass concentration $\left(X\right)$ was calculated using the following Eq. 1:

$$X\;\left(g\;L^{-1}\right)=\frac{m_{fb}-m_{f}}{V}$$ (1)

Where $m_{fb}$ is the mass of the dried filter and biomass together (g), $m_{f}$ is the pre-dried void filter mass and V is the sample filtered volume (L).

##### 2.3.2.2 Chlorophyll content

Chlorophyll content was quantified in each bioassay every 3 days to continue evaluating native MBC growth performance in NSWW and SWW, following Pompelli *et al.* approach (Pompelli et al., 2013). Briefly, 2 mL of the sample was centrifuged at 4000 rpm for 20 min and then the concentrated pellet was homogenized with the addition of acetone (90% v/v); which consequently, was placed into an ultrasonic bath at 5°C for 60 min. Samples remained stored at 0°C for 12 h to complete the extraction of chlorophyll and to facilitate the separation of the chlorophyll-containing supernatant during the subsequent centrifugation step (Hosikian et al., 2010). Later, samples were centrifuged at 4000 rpm for 20 min and the supernatant’s absorbances at 664 and 647 nm wavelength were measured, respectively.

The total chlorophyll content (Chl_T_) was calculated using the following equation (Pompelli et al., 2013):

$$Chl_{T}\left(g\;L^{-1}\right)=6.43A_{664}+18.43A_{647}$$ (2)

Where $A_{664}$ and $A_{647}$ are the absorbances measured at wavelengths 664 and 647 (nm), respectively.

##### 2.3.2.3 Nutrient removal rates and efficiencies

Nutrients (NH_4_^+^-N, NO_3_^−^-N, and PO_4_^3-^-P) and total and soluble COD concentrations were quantified in each bioassay every 3 days to evaluate the domestic wastewater bioremediation performance of the native MBC.

The nutrient removal rates (RR) were calculated from the slope of the concentration as a function of time using the following equation (Benítez et al., 2019):

$$RR\left(mg\;L^{-1}d^{-1}\right)=\frac{C_{o}-C_{f}}{t_{f}}$$ (3)

Where $C_{o}$ and $C_{f}$ are the nutrients’ initial and final concentrations (mg L^-1^) and $t_{f}$ is the time frame (days).

The nutrient removal efficiencies (RE) were calculated using the following equation (Benítez et al., 2019):

$$RE\left(\%\right)=\frac{C_{o}-C_{f}}{C_{o}}*100$$ (4)

Where $C_{o}$ and $C_{f}$ are the nutrients’ initial and final concentrations (mg L^−1^).

### 2.4 Statistical analysis

Experimental data were evaluated using Minitab Statistical Software (version 21.1.0) for two-paired samples two-tailed *t*-Test for determining significant differences in (1) wastewater’s physico-chemical average characterization before and after sterilization, and (2) X and Chl_T_ content in NSWW and SWW, followed by a two-way analysis of variance (ANOVA) and a pairwise Tukey’s *post hoc* at a significance level of 0.05 to test the effect of wastewater type (NSWW, SWW) and Ecuadorian Amazon’s native MBC (M1, M2, M3, M4, M5, M6) in terms of organic matter and nutrients removal rates expressed as mg L^−1^ d^−1^. The error reported for all values indicates one standard deviation.

## 3 Results and discussion

### 3.1 Domestic wastewater characterization

Table 1 shows the average composition of 3 composite samples of domestic NSWW and SWW collected from the wastewater discharge point at Universidad San Francisco de Quito (Cumbayá, Ecuador) from October 2018 to January 2019. NSWW and SWW samples were statistically different in their concentration of NH_4_^+^-N, PO_4_^3-^-P, total and soluble COD (sCOD), and BOD; as well as their pH, T, and EC values. The concentrations of sulphate and sulfide were within the national guidelines and did not significantly differ between NSWW and SWW (TULSMA, 2015). As such, these two parameters were not expected to influence the efficiency of the microalgal-bacterial treatment system during the nutrient removal bioassays in the presence of non-sterilized and sterilized wastewater samples evaluated in this study.

**TABLE 1:** Physico-chemical average characterization of NSWW and SWW domestic wastewater collected from October 2018 to February 2019.

Parameter	Unit	NSWW	SWW	p-value
Ammonium (NH_4_^+^-N)	mg L^−1^	259.03 ± 7.06	117.37 ± 4.78	0.07
AmandNitrate (NO_3_^−^-N)	mg L^−1^	0.12 ± 0.01	0.13 ± 0.01	0.23
Phosphate (PO_4_^3-^-P)	mg L^−1^	30.78 ± 3.55	24.88 ± 4.05	0.02
Total COD (COD)	mg L^−1^	777.87 ± 40.43	653.97 ± 29.70	0.04
Soluble COD (sCOD)	mg L^−1^	620.39 ± 47.61	428.23 ± 41.77	0.01
BOD_5_	mg L^−1^	228.89 ± 15.40	87.78 ± 5.77	0.04
Chloride (Cl^−^)	mg L^−1^	135.84 ± 11.16	135.12 ± 12.58	0.45
Fluoride (F^−^)	mg L^−1^	0.70 ± 0.20	0.68 ± 0.13	0.42
Sulphide (S^2-^)	mg L^−1^	0.32 ± 0.09	0.23 ± 0.02	0.14
Sulphate (SO_4_^2-^)	mg L^−1^	36.18 ± 2.70	26.08 ± 1.42	0.08
TSS	mg L^−1^	0.21 ± 0.03	0.14 ± 0.02	0.21
Dissolved oxygen (DO)	mg L^−1^	0.48 ± 0.02	1.48 ± 0.00	0.13
pH	-	7.29 ± 0.15	7.74 ± 0.15	0.04
Temperature (T)	°C	19.99 ± 0.07	22.40 ± 0.09	0.00
Conductivity (EC)	µS cm^−1^	1116.44 ± 23.82	999.75 ± 0.70	0.01

Relative to NSWW, the compositional and physico-chemical differences observed for SWW could be due to the application of high temperature throughout the sterilization process. SWW was subject to a temperature of 121°C and saturated steam under 15 psi of pressure for 30 min in the autoclave to deactivate any autochthonous microorganisms and bacterial spores present in the collected wastewater (Safitri et al., 2021).

Total and soluble COD concentrations were lower in SWW (653.97 ± 29.70 and 428.23 ± 41.77 mg L^-1^) relative to NSWW (777.87 ± 40.43 and 620.39 ± 47.61 mg L^-1^) plausibly because the autoclave’s pressurized steam enhances volatilization and oxidation of the wastewater’s originally present organic compounds, often having low boiling points and high vapor pressures (Feng et al., 2021). Furthermore, the application of steam on microorganisms could cause artefactual physical damage to their cells (Rezaiyan and Cheremisinoff, 2005). This reduction of organic matter after wastewater sterilization not only resulted in a lower total and sCOD concentration, but also in a lower BOD_5_ concentration in SWW compared to NSWW by a factor of 2.61 due to the positive correlation that exists between these two parameters (Rai et al., 2019).

NH_4_^+^-N concentration was higher in NSWW than in SWW by a factor of 2.21. We speculate that the high temperatures reached in the autoclave process increase the conversion rate of dissolved NH_4_^+^ to ammonia gas (NH_3(g)_). When temperature rises, CO_2_ solubility in wastewater decreases causing CO_2(g)_ to be expelled leaving a more alkaline pH (Adnan et al., 2020). Under these physicochemical conditions, the disassociation of NH_4_^+^ ions is increased, and the equilibrium between NH_3_ liquid and gas phase shifts towards generating NH_3(g)_ that leaves the wastewater sample (Mohammed-Nour et al., 2019).

PO_4_^3-^-P concentration was also lower in SWW than in NSW likely due to the high temperatures reached during the autoclave process, which plausibly led to the precipitation of dissolved PO_4_^3-^ (González-Morales et al., 2021). As all physicochemical characterization measurements were conducted in filtered samples, it is possible that PO_4_^3-^ precipitates remained trapped in the filter. A study conducted with similar autoclaved conditions (Padri et al., 2022) also reported post-sterilization reduced media content of PO_4_^3-^-P and COD from 54.1 ± 3.21 and 205 ± 12.3 mg L^-1^ to 20.14 ± 0.64 and 178 ± 4.64 mg L^-1^, respectively.

Nutrients such as NH_4_^+^-N and PO_4_^3-^-P also contribute to the conductivity values of the wastewater (Levlin, 2007). Thereby, the reduction of these ions content described above explains the reason why SWW presented a lower conductivity value when compared to that of NSWW, 999.75 ± 0.70 versus 1116.44 ± 23.82 μS cm^−1^, respectively.

### 3.2 Growth response of Ecuadorian Amazon’s microalgal-bacterial consortia (MBC) to wastewater

**FIGURE 1:**
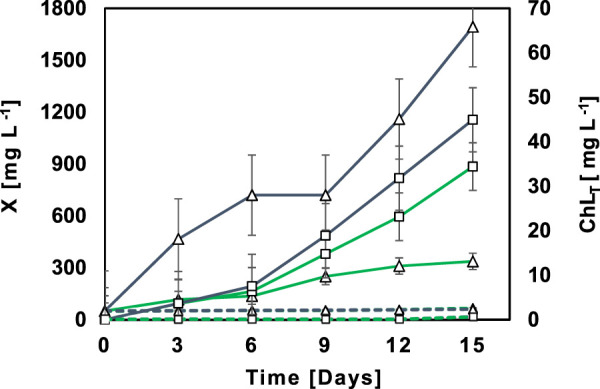
Profiles of biomass (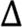) and total chlorophyll (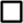) evolution along 15 days of cultivation in NSWW (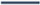) and SWW (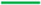) for the treatment bioassays (T5) with microalgal-bacterial consortia M5 and abiotic controls in NSWW (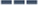) and SWW (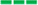), respectively.

An illustrative example of the growth of native microalgal-bacterial consortia (MBC) M5 in NSWW and SWW is presented in Figure 1. Consortia growth -measured as biomass accumulation-in NSWW (136.94 mg L^-1^ d^−1^) was significantly higher (*p* = 0.01) than in SWW (21.69 mg L^−1^ d^−1^). Growth patterns for NSWW and SWW were different. In SWW, the lag phase lasted for 6 days followed by a short exponential growth phase that peaked on day 9 and then remained in the stationary phase until the end of the experiment (337 mg L^−1^ at day 15). Conversely, in NSWW, MBC directly entered an exponential growth phase from day 0 that endured until the end of the experiment (1693 mg L^-1^ at day 15). The same trend was observed with all MBC evaluated in this study. Similar biomass accumulation trends were presented by Ma et al. (2014). In the aforementioned study, microbial growth in SWW started with a lag phase for 2 days, followed by the exponential growth phase that peaked on day 3 (680 mg L^-1^), and finalized in a stationary phase that lasted from day 3 onwards (day 7). In NSWW, there was no lag phase, and the stationary phase, following the exponential growth phase that peaked on day 2 (1170 mg L^-1^), lasted from day 5 onwards (day 7).

The profiles in Figure 1 suggest that Ecuadorian MBC have a greater adaptation to and performance in NSWW, this could be attributed to (1) differences in nutrient and organic matter composition between NSWW and SWW and/or (2) possible synergy between the autochthonous microbial community substrates/metabolites in the wastewater and the native MBC. Noteworthy, in the control assays, AC containing NSWW and SWW (Figure 1), a negligible growth (0.60 mg L^−1^ d^−1^) was observed.

Regarding the difference in media composition, in SWW, the sterilization process depleted the entire active autochthonous microbial community and potentially eliminated growth-promoting dynamics and metabolic interactions. Likewise, the availability of adequate nutrients’ quantity in SWW was diminished, inducing cellular stress in the MBC which could prolong the lag phase and cause a low rate of exponential growth (Hamill et al., 2020). After autoclaving, the COD of the wastewater effluents dropped from 620.39 ± 47.61 to 428.23 ± 41.77 mg L^−1^ (Table 1), thus restricting organic carbon sources for bacterial respiration geared to growth and cell division. With less bacteria producing CO_2_ and inorganic substances, microalgal growth can be affected. Padri et al. (2022) investigated the co-culture of *C. sorokiniana* and *Streptomyces thermocarboxydus* in unsterilized/sterilized wastewater. They exhibited germination and growth limitations when the carbon source was lower than 500 mg L^−1^. Table 1 shows that further fundamental macronutrients in the form of PO_4_^3-^—P and NH_4_^+^- N declined in like manner from 30.78 ± 3.55 and 259.03 ± 7.06 mg L^−1^ in NSWW to 24.88 ± 4.05 and 117.37 ± 4.78 mg L^−1^ in SWW, respectively. Salem *et al.* (Salem et al., 2013) reported that < 30 mg P L^−1^ of K_2_HPO_4_ induces *Phormidium sp.* growth stress and An *et al.* (An et al., 2020) observed that < 246 mg N L^−1^ of CH_4_N_2_O lowers *Chlorococcum ellipsoideum*’*s* growth rate. As explained in Yaakob *et al.* review (Yaakob et al., 2021), a deficiency of P and N in SWW inhibits cell growth drastically by N depletion affecting ATP synthesis while degenerating thylakoid membrane-rich chloroplasts and P deficiency causing deformations of chloroplast and mitochondria. Our study revealed that the sterilization process led to a decline in crucial macronutrients, such as organic carbon, phosphate, and ammonia, which are essential for microbial growth.

Possible interactions of the Ecuadorian native MBC with the autochthonous microbial community could also prompt biomass growth immediately (absence of lag phase) along with such an elongation of the exponential growth phase in NSWW amid the experimental period. Certain bacteria have been found to promote microalgal growth in wastewater by creating a favorable microenvironment for multiple microalgal species such as *Azospirillum brasilense* for *Chlorella vulgaris*, *Rhizobium sp*. for *Chlamydomonas reinhardtii* and *Stappia sp.* for *Tetraselmis striata* (Toyama et al., 2019). As elucidated in Palacios *et al.*’s review (Palacios et al., 2022), microalgae growth-promoting bacteria (MGPB) possess two principal mechanisms to boost microalgal growth and metabolism: i) production of exogenous phytohormone related compounds (gibberellins, cytokines, and auxins) and ii) production of co-factors and compounds to mitigate environmental stress (cobalamin and riboflavin vitamins). In return, these primary producers support heterotrophic bacteria growth by supplying oxygen (O_2_) as an electron acceptor for organic matter degradation (Acién et al., 2016). Nevertheless, autotrophic microalgal activity ceases under dark conditions, mixotrophic activities take place and bacterial activity continues ascribed to the remaining DO ruled by microalgae photosynthesis at daytime. Mhedhbi *et al.* (Mhedhbi et al., 2020), demonstrated uninterrupted nocturnal bacteria’s metabolic functions by detecting a significant decrease in the DO rate at night (24-30 h) in PBRs containing municipal wastewater. In addition, the better performance of microalgal-bacteria consortia in NSWW compared to SWW could be also attributed to HDB. This rudimentary behavioral phenomenon is believed to diminish cells’ time needed to adjust when a similar environment re-occurs (Cerulus et al., 2018). Therefore, it is likely that Ecuadorian Amazon’s water bodies from where microalgal-bacterial consortia (MBC) were collected, experienced domestic wastewater contamination by surrounding towns. The release of untreated domestic wastewater to freshwater bodies is a common practice in Ecuador due to the lack of wastewater treatment plants in the country (Maurice et al., 2019).

In addition to biomass content, Chl_T_ also acted as an indicator of active growth and adaptation potential, although exclusively for microalgae using Eq. 2. The illustrative example of MBC M5 in Figure 1 corroborates that Ecuadorian Amazon’s microalgae growth in NSWW (Chl_T_ = 3.08 mg L^−1^ d^−1^) was significantly higher (*p* = 0.00) than in SWW (Chl_T_ = 1.34 mg L^−1^ d^−1^). In fact, the final Chl_T_ content (Day15) measured in NSWW and SWW were ranked in the order of 44.95 mg L^−1^ (WW) > 34.42 mg L^−1^ (SWW), indicating a more successful bioconversion of wastewater’s nutrients and CO_2_ into microalgae biomass when cultivated in NSWW.

Goswami et al. (2019) and Ge et al. (2018), also used Chl_T_ to monitor microalgae growth in municipal wastewater. They reported final Chl_T_ contents of 21.87 and 10.20 mg L^−1^ for a 2:2 microalgal-bacterial consortia in non-sterilized wastewater and an axenic *Chlorella vulgaris* culture in sterilized wastewater, respectively. It is worth noting that in Figure 1, ACs containing NSWW or SWW respectively, indicated zero Chl_T_ content until the end of the experiment which suggests that the domestic wastewater did not hold microorganisms able to carry this photosynthetic pigment or those microorganisms were not active. Henceforth, native microalgal-bacterial consortia from the Ecuadorian Amazon Region seemed to be the sole source of photoautotrophic microorganisms in the treatment bioassays (Ts).

### 3.3 Macronutrient removal patterns from MBC native to the Ecuadorian Amazon

The initial and final concentrations of sCOD, NH_4_^+^-N, NO_3_^−^-N, and PO_4_^3-^-P in each bioassay conducted with NSWW and SWW are presented in Supplementary Table S1. As expected, the initial concentrations of organic matter and nutrients were higher in NSWW compared to SWW due to the difference in media composition, while the final concentrations varied depending on the performance and metabolic activity of each MBC as described below.

Every treatment in NSWW and SWW cultivated with native MBC was compared to the abiotic controls (lacking native MBC), which underwent the same analytical procedures. The statistical analysis (Table 2) showed significant differences in removal rates (RRs) calculated using Eq. 3 of C, P, and N between Ts and AC (Tukey’s test; *p* < 0.05), thus validating the predominance of biological uptake of macronutrients from wastewater in nearly all PBRs. The only one to deviate from this trend was T5 (NO_3_^−^-N, RR = 0.01 ± 0.00 mg L^−1^ d^−1^) which ended up not being statistically different from the AC (NO_3_^−^-N, RR = 0.00 ± 0.00 mg L^−1^ d^−1^). From these results, we suspect that MBC M5 did not have microorganisms capable of denitrification; though more research is needed on the consortium’s microbial communities’ taxonomic and functional profiles that remain unknown.

**TABLE 2:** Post hoc Tukey’s test results for RR of sCOD, NH_4_^+^ -N, NO_3_^−^ -N and PO_4_^3-^ -P between 6 native microalgal-bacterial consortia (MBC) from the Ecuadorian Amazon and the abiotic controls in NSWW and SWW. Bioassays marked with the same letters are not significantly different ( α = 0.05).

Bioassay	sCOD [mg L^-1^d^-1^]	NH_4_^+^-N [mg L^-1^d^-1^]	NO_3_^−^N [mg L^-1^d^-1^]	PO_4_^3—^P [mg L^-1^d^-1^]
AC	B	B	D	B
T1	A	A	CD	A
T2	A	A	A	A
T3	A	AB	ABC	AB
T4	A	AB	AB	A
T5	A	A	D	A
T6	A	AB	BCD	A

**FIGURE 2:**
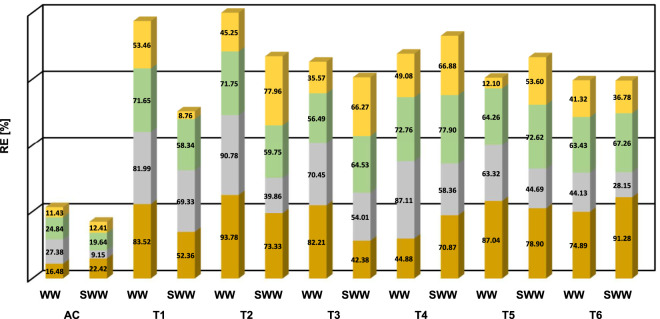
Removal percentage’s profiles (RE) of (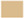) sCOD (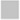), NH_4_^+^-N (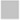), PO_4_^3-^-P and (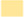) NO_3_^−^ -N along 15 days of cultivation in NSWW and SWW with MBC M1, M2, M3, M4, M5 and M6 in T1, T2, T3, T4, T5, T6, respectively and abiotic controls (AC).

Despite all six MBCs being able to remove C, N, and P from domestic wastewater, Figure 2 reveals that i) some consortia perform better than others and ii) the performance was better in NSWW than in SWW. MBC M2, M1, and M4. exhibited the highest removal efficiencies (RE) calculated using Eq. 4 for organic matter and nutrients in NSWW. MBC M2 was the frontrunner in COD (93.78% ± 0.57%) and NH_4_^+^-N (90.78% ± 3.14%) removal, followed by M1 (COD = 83.52 ± 0.14%, NH_4_^+^-N = 81.99 ± 4.62%) and M4 (COD = 44.84 ± 7.34%, NH_4_^+^-N = 87.11 ± 4.11%). The latter showed superior PO_4_^3-^-P uptake (72.76% ± 8.97%), followed by M2 (71.75% ± 2.73%), and M1 (71.65% ± 2.29%). M1 led the other consortia in NO_3_^−^-N removal (53.46% ± 8.56%), followed by M4 (49.08% ± 10.57%) and M2 (45.25% ± 11.35%). These differences in performances could be attributed to the existing diversity in each consortium, which is highly influenced by their original environment (Shen et al., 2021). As is depicted in Supplementary Figure S1, M1, and M2 were further away (>4 km) from Lago Agrio’s urban parish (Nueva Loja) than the remaining consortia (<2 km). Hence, by being more immersed in rural parishes, it is likely that the natural habitat of T1 and T2 was more pristine and less affected by anthropogenic activities.

It is important to notice that recognizing M2, M1, and M4 as the optimum performing MBC for wastewater treatment is based on their ability to remove nutrients and organic matter in NSWW exclusively. In practice, RE under sterilized conditions (SWW) is operationally inefficient at scale (Geremia et al., 2021). Notwithstanding, at a laboratory scale analyzing SWW along NSWW provided insights into the influence of the difference in media composition (sterilized vs. non-sterilized) -including the plausible influence of the autochthonous microbial community—on the remediation efficiency of MBC. At first glance in Figure 2, more than half of the consortia seem to perform better in NSWW than SWW in terms of the RE for sCOD, NH_4_^+^-N. Noteworthy, 4 out of the 6 MBCs presented the highest RE for sCOD in NSWW (82.21%–93.78%) compared to the values in SWW (42.38%–78.90%). In the case of NH_4_^+^-N, the same trend was observed for all consortia evaluated in this study, higher RE were obtained in NSWW (44.13%–90.78%) versus SWW (28.15%–69.33%). For PO_4_^3-^-P uptake, MBC M1 and M2 performed better in NSWW than in SWW (71.65% and 71.75% versus 58.34% and 59.75%) whereas there was no difference between the performance of M4 and M6 in NSWW and SWW. Finally for NO_3_-N, the removal efficiencies were higher for M1 and M6 in NSWW (53.46% and 41.32%) compared to SWW (8.76% and 36.78%). Our results are in agreement with literature studies on the removal efficiencies of nutrients and organic matter using microalgal-bacterial consortia. For instance, Najafi Chaleshtari *et al.* reported removal efficiencies of ammonium and phosphate of 94.36 + 3.5% and 88.37 + 3%, respectively, using a microalgal activated sludge membrane bioreactor cultivated in raw wastewater (Najafi et al., 2022). Similarly, Moondra et al. achieved removal efficiencies of 97.40% for phosphate, 94.05% for ammonia, and 88.40% for COD with raw domestic wastewater under 8- and 16-h HRTs (Moondra et al., 2021).

Table 3 presents the RRs for sCOD, NH_4_^+^-N, NO_3_^−^-N, and PO_4_^3-^-P of the 6 native MBCs from the Ecuadorian Amazon cultivated in NSWW and SWW. Changes in macronutrients’ RRs (Table 3) and concentrations (Figure 2) are thoroughly evaluated below to outline their possible metabolic routes and fate in the 15 days of evaluation.

**TABLE 3:** RR of sCOD, NH_4_^+^-N, NO_3_^−^-N and PO_4_^3-^-P of 6 native microalgal-bacterial consortia (MBC) from the Ecuadorian Amazon cultivated in NSWW and SWW.

		sCOD [mg L^-1^d^-1^]	NH_4_^+^-N [mg L^-1^d^-1^]	NO_3_^−^N [mg L^-1^d^−^]	PO_4_^3-^-P [mg L^-1^d^-1^]
Wastewater type	Bioassay				
**NSWW**	AC	6.85 ± 0.85	3.39 ± 0.51	0.00 ± 0.00	0.91 ± 0.45
	T1	37.13 ± 0.62	9.24 ± 1.44	0.14 ± 0.02	7.21 ± 0.42
	T2	33.66 ± 1.30	12.43 ± 2.69	0.17 ± 0.07	7.52 ± 0.33
	T3	38.27 ± 7.83	6.62 ± 0.50	0.12 ± 0.03	2.72 ± 0.18
	T4	38.24 ± 11.52	7.34 ± 1.33	0.31 ± 0.10	8.32 ± 1.45
	T5	32.90 ± 1.20	6.68 ± 0.20	0.01 ± 0.00	6.74 ± 0.20
	T6	38.30 ± 2.46	6.41 ± 0.24	0.10 ± 0.03	5.09 ± 1.37
**SWW**	AC	8.87 ± 1.50	0.51 ± 0.44	0.02 ± 0.01	0.55 ± 0.22
	T1	62.59 ± 12.7	4.79 ± 0.16	0.01 ± 0.01	4.34 ± 0.74
	T2	70.89 ± 13.8	4.71 ± 0.65	0.60 ± 0.15	3.91 ± 0.15
	T3	41.96 ± 9.0	2.91 ± 0.24	0.29 ± 0.01	4.55 ± 0.66
	T4	74.96 ± 11.3	3.89 ± 0.97	0.21 ± 0.08	2.55 ± 0.17
	T5	86.28 ± 5.60	6.17 ± 1.71	0.08 ± 0.00	6.16 ± 0.60
	T6	60.48 ± 10.1	2.34 ± 1.06	0.08 ± 0.01	4.42 ± 0.72
**NSWW and SWW Interaction p-value**		0.04	0.00	0.00	0.00
**WW and SWW Tukey’s Post hoc**		AB	CD	EF	GG

#### 3.3.1 Carbon removal

**FIGURE 3:**
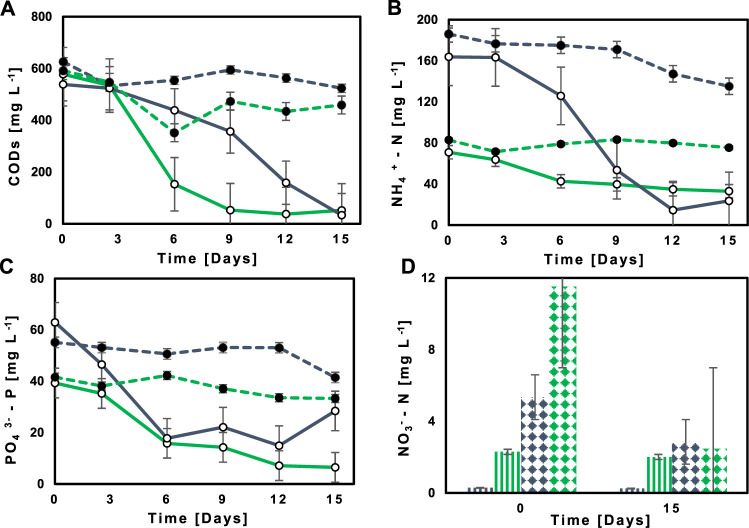
Concentration’s profiles of **(A)** COD, **(B)** NH_4_^+^-N, **(C)** PO_4_^3-^-P along 15 days of cultivation in NSWW (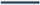) and SWW (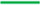) in T2 (M2) (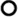) and abiotic control (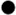). **(D)** Concentration profile of NO_3_^−^-N in NSWW (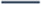) and SWW (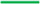) for consortium T2 (M2) (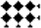) and the abiotic control (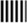) at day 0 and also day 15.

The illustrative example in Figure 3A depicts how Ecuadorian Amazon’s native M2 reduced the concentration of sCOD very efficiently from 538.44 to 578.06 mg L^−1^ to 33.47 and 51.92 mg L^−1^ in NSWW and SWW, respectively. The same trend was observed for all MBC evaluated in this study. The treated effluent’ sCOD was within the limits of national discharge guidelines as established in the national legislation (TULSMA, 2015), whether compared to the maximum permissible limit of 250 mg L^−1^ for disposal into the public sewerage system or 500 mg L^−1^ into freshwater bodies. Notwithstanding, compliance with the more restrictive value is preferred in this study since rivers are the ultimate sink for the 96.5% of untreated wastewater generated in Quito, Ecuador (Borja-Serrano et al., 2020).

With respect to the performance of the consortia, Table 3 presents the sCOD RR in NSWW and SWW. In the case of sCOD RR in NSWW, the values were very similar for the 6 MBC evaluated in this study, ranging from 32.90 ± 1.2 (M5) to 38.30 ± 2.46 mg L^−1^ d^−1^ (M6); while the sCOD RR in SWW varied from 6.59 ± 12.7 (M1) to 86.28 ± 5.6 mg L^−1^ d^−1^ (M5). In general terms, the sCOD RR was significantly higher (*p* = 0.04) by 56.12% when shifting from NSWW (36.42 ± 4.16 mg L^−1^ d^−1^) to SWW (56.86 ± 10.42 mg L^−1^ d^−1^) on average. It is interesting to notice that this data is challenged by the growth profiles of the MBC in Figure 1, as microalgal-bacterial population enlargement was wider in NSWW relative to SWW. It is widely acknowledged that as heterotrophic bacteria are the prime actors in C removal then an increase in bacterial population must come with a parallel COD elimination (Perera et al., 2022). Previous studies have reported higher COD RRs in non-sterilized wastewater by virtue of the mutualistic interactions alongside autochthonous microbial community promoting bacterial respiration (Geremia et al., 2021; Mohsenpour et al., 2021; Yang et al., 2022). Likewise, microalgae in wastewater seem to excrete more organic C in the form of extracellular polymer substances (EPS) that could enhance wastewater’s COD (Lee et al., 2019). Therefore, it is likely that the carbon RR of the MBC was load dependent, thus it is possible that under the conditions evaluated in this study, it was decreasing in non-sterile conditions because of bacteria having to decompose a higher load of microalgae-produced COD. Microalgae are known for their efficient carbon fixation processes and biomass production (Chan et al., 2022).

#### 3.3.2 Nitrogen removal

The illustrative example in Figure 3B indicates how NH_4_^+^- N in NSWW and SWW is reduced to 15 and 33 mg L^−1^ from initial concentrations of 164 and 71 mg L^−1^, respectively. Since NH_4_^+^- N is not a normalized parameter by the Ecuadorian legislation, it was compared to international regulations. Belarus’s Technical Code and German’s Ordinance AbwV specify the limit of NH_4_^+^-N in wastewater discharged into receivers at levels not exceeding 15–20 and 10 mg L^−1^, respectively (Preisner et al., 2020). When compared to the first one, the concentration in the treated effluent reported in this study fell below the limit.

Research on the wastewater treatment of NH_4_^+^- has identified microalgal biomass assimilation and nitrification as the two main biological removal mechanisms. Figure 1; Figure 3D depict how NO_3_^−^-N concentration increased at day zero from 5.35 to 11.51 mg L^−1^ and microalgal biomass (Chl_T_) decreased from 3.08 to 1.34 mg L^−1^ d^−1^ in NSWW and SWW, respectively. Based on these results, it is possible that the fate of NH_4_^+^-N in NSWW was mainly microalgal biomass, whereas in SWW it was oxidized N. Under non-sterile conditions, microalgae played the main role on account for a larger NH_4_^+^-N (164 mg L^−1^) availability and could have received support from satellite bacteria belonging to the consortia themselves in the WW effluent. It has been well documented that MGPB boosts the activities of both enzymes: glutamine synthetase (GS) and glutamate dehydrogenase (GDH) in microalgae’ NH_4_^+^-N metabolic pathway which could be 2-fold higher than nitrification (González-González and de-Bashan, 2021). In fact, at NH_4_^+^-N concentrations like the starting one (164 mg L^−1^), several studies have reported microalgae repressing nitrification by 77%; albeit not entirely (Fallahi et al., 2021). Therefore, it is probable that the autochthonous microbial community in the wastewater also acted as a supplement for the nitrifying populations in the MBC consortia to partly overcome this restraint. Literature studies report that domestic wastewater represents an important seeding of active ammonia oxidizing bacteria (AOB) and nitrite oxidizing bacteria (NOB) (Zeng et al., 2014).

Since assimilation into microalgal biomass is dominant in NSWW, the removal rate of NH_4_^+^-N (8.04 ± 1.07 mg L^−1^ d^−1^) in NSWW was significantly higher (*p* = 0.00) than in SWW (4.14 ± 0.75 mg L^−1^ d^−1^) (Table 3). The NH_4_^+^-N RR in NSWW ranged from 6.41 ± 0.24 (M6) to 12.43 ± 2.69 mg L^−1^ d^−1^ (M2) while the RR values in SWW were considerably lower, varying from 2.34 ± 1.06 (M6) to 6.17 + 1.71 mg L^−1^ d^−1^ (M5). In this last NH_4_^+^-N limiting environment (71 mg L^−1^) (Figure 3B), it is likely that AOB and NOB repressed microalgal activity due to competition (González-Camejo et al., 2020), consequently, positioning nitrifies as main responsible for NH_4_^+^-N uptake. Along these lines, González *et al.* (González-Camejo et al., 2020) also demonstrated that NO_3_^−^-N concentration increased from 1.3 to 17.5 mg L^−1^ under replete and deplete NH_4_^+^-N conditions, respectively, for a microalgae-nitrifying bacterium consortium cultured in Valencia Carraixet’s wastewater effluents. As NO_3_^−^-N load increased in wastewater, the necessity to treat it increased as well. In SWW more urgently than in NSWW, since its initial concentration of 11.51 mg L^−1^ (Figure 3D) surpassed the national maximum discharge limit (NO_3_^−^-N < 10 mg L^−1^) for freshwater bodies (TULSMA, 2015). The illustrative example in Figure 3D provides evidence of how efficiently the Ecuadorian Amazon MBC M2 removed NO_3_^−^-N in SWW and NSWW to 2.46 and 2.86 mg L^−1^, respectively. When compared to the national guidelines, the post treatment concentrations of the wastewater were far below the value allowed by the national legislation. It should be highlighted that NO_3_^−^ -N concentrations may be underestimated owing to the occurrence of simultaneous denitrification (Foladori et al., 2020). Research on the wastewater treatment of NO_3_^−^ -N has identified heterotrophic and autotrophic denitrification as the two main biological removal mechanisms (Zhang et al., 2022). When NH_4_^+^-N is readily available, microalgae use it as the main N source (Li et al., 2019). Indeed, autotrophic microorganisms have a preference for NH_4_^+^-N intake given the low metabolic cost for reducing it to organic matter as it is directly incorporated into amino acids and proteins via GS-GOGAT cycle by glutamine synthetase (GS)-glutamate synthase (GOGAT) enzymes (Salbitani and Carfagna, 2021). Conversely, NO_3_^−^-N assimilation demands microalgae to prior reduce it to NO_2_ in cytosol and then to NH_4_^+^-N in chloroplast’s stroma (Pozzobon et al., 2021). Consequently, heterotrophic denitrification is more likely to occur in PBR’s. However, this does not rule out the possibility of microalgae supporting bacterial denitrification by excreting transparent exopolymer particles (TEP) and EPS thus providing supplementary sources of mineralizable C (Laverman et al., 2021).

Table 3 shows that the NO_3_^−^-N RR in NSWW and SWW varied from 0.01 ± 0.01 (M1) to 0.31 ± 0.10 mg L^-1^ d^-1^ (M4 in NSWW) and to 0.60 ± 0.15 mg L^-1^ d^-1^ (M2 in SWW). However, according to Tukey’s *post hoc* test (Table 2), despite existing interaction (*p* < 0.05) between the type of wastewater and MBC; the means of NO_3_^−^-N RR in NSWW (0.14 ± 0.04 mg L^−1^ d^−1^) were not significantly different (GG) than the ones obtained in SWW (0.21 ± 0.04 mg L^−1^ d^−1^). This is expected on account of both environments having similar DO concentrations varying within 1.02–3.02 mg L^−1^ in SWW and 1.86—4.64 mg L^−1^ in NSWW. Nonetheless, what is even more remarkable is that NO_3_^−^-N elimination by biological modulation was carried out under aerobic conditions. In the present study, microalgal photosynthesis was constantly generating O_2,_ and the remainder was provided uninterruptedly with a diffuser aeration of 1 L s^−1^ using an air pump. In geometries like PBRs, evolved and supplemented O_2_ easily swell up to high concentrations (Kazbar et al., 2019). Therefore, as O_2_ was readily available at all times, it was unlikely that the traditional low-or no-O_2_ pathway for NO_3_^−^-N removal occurred in either of our experimental conditions. Wang et al. (2021) reported that increasing DO from 1 mg L^−1^ hereinafter gradually suppressed the microbial anaerobic NO_3_^−^-N respiration activity during the wastewater treatment.

In consequence, our data suggests that aerobic denitrification was the predominant NO_3_^−^-N biological metabolic pathway in the present study, thus possessing a unique cost-operational advantage of allowing simultaneous nitrification and denitrification in one single aerated reactor (Yang et al., 2020). Of note in Table 1, the original NO_3_^−^-N concentration in the domestic wastewater effluents evaluated in this study (SWW = 0.13 mg L^−1^ and NSWW = 0.12 mg L^−1^) did not really require treatment for being innocuous to hydrobionts and humans (Al-Housni et al., 2020; Shinoda et al., 2021).

#### 3.3.3 Phosphorus removal

Table 3 shows that PO_4_^3^ -P on average is removed significantly higher (*p* = 0.00) in NSWW (6.27 ± 0.66 mg L^−1^ d^−1^) compared to SWW (4.32 ± 0.51 mg L^−1^ d^−1^). The range of PO_4_^3-^ -P RR in NSWW were between 2.72 ± 0.18 (M3) and 7.52 ± 0.33 mg L^−1^ d^-1^ (M2) and in SWW the values ranged between 2.55 ± 0.17 (M4) and 6.16 ± 0.60 mg L^−1^ d^−1^ (M5). The better performance of MBC in NSWW could be attributed to the presence of autochthonous microorganisms in the wastewater and the composition of the wastewater as described above. As illustrated by Sial et al. (2021), *E. coli* furnish microalgae with inorganic P by decomposing P-containing matter commonly found in wastewater. In conjunction with the inorganic P naturally present in wastewater’s proteins, lipids, and nucleic acids autotrophs will then start PO_4_^3-^-P translocation across cells’ plasma membrane and phosphorylation involving 3 adenosine diphosphate (ADP) to synthesize energy storage molecules (3 ATP and 2 NADPH) (Chai et al., 2021). Additionally, wastewater is a source of PO_4_^3-^-P-accumulating organisms (PAO), *Candidatus Accumulibacter phosphatis* bacteria in particular (Begmatov et al., 2022), capable of taking up PO_4_^3-^-P from their immediate environment and accumulating it as polyphosphate (Poly-P) fueled by their stored polyhydroxyalkanoates (PHAs) under aerobic conditions (Chu et al., 2022; Klein et al., 2022).

**FIGURE 4:**
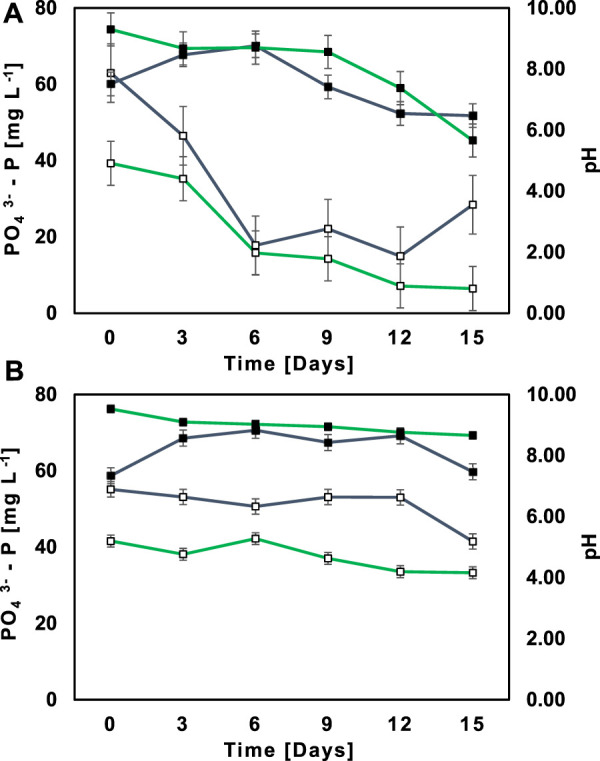
Profiles of PO_4_^3-^-P concentration (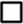) and pH measurement (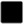) along 15 days of cultivation in NSWW (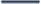) and SWW (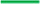) for native MBC T2 **(A, B)** abiotic control.

The illustrative example in Figure 3C depicts Ecuadorian Amazon’s native MBC M2 achieving lower PO_4_^3-^-P concentrations in SWW (6.47 mg L^−1^) compared to NSWW (28.45 mg L^−1^). This data suggested that besides PO_4_^3-^-P being assimilated by microorganisms; it was also chemically precipitated under sterile circumstances (Larsdotter et al., 2007). Several research studies (Cerozi and Fitzsimmons, 2016; Wang et al., 2022) revealed that under alkaline conditions (8.5 < pH < 10.5) most dissolved free PO_4_^3-^-P species form insoluble compounds (e.g., hydroxyapatite and octocalcium phosphate) that decrease PO_4_^3-^-P availability in aqueous solutions and induce its deposition (Lei et al., 2021). Such chemical stripping given by pH variations in wastewater is biologically mediated by C and N assimilation. In fact, nitrification decreases pH; meanwhile denitrification and decomposition increase pH (Deng et al., 2021). In the present study, the RR of NH_4_^+^-N was lower; while in the case of NO_3_^−^-N and sCOD, RR was higher in SWW compared to NSWW (Table 3). Therefore, pH tended to rise much more and achieved greater values in SWW (7.38 < pH < 9.30) *in lieu* NSWW (6.54 < pH < 8.70) within days 0–12, as shown in Figure 4A. Consequently, the illustrative example profiles lower PO_4_^3-^-P concentrations achieved in SWW regarding of larger precipitation followed by filtration *a priori* PO_4_^3-^ -P measurement in a laboratory. The same exact pattern followed the AC in Figure 4B although with less variation in pH and PO_4_^3-^-P concentrations over time due to the absence of M2. These results are comparable to those from Beltran *et al.* (Beltrán-Rocha et al., 2021) who observed a non-biological P salt precipitation along with a pH increase in microalgal-bacterial consortia (Prazeres et al., 2021).

PO_4_^3-^-P is not a normalized parameter in the national legislation, so the values were compared to Switzerland’s legal framework “Federal Water Protection Law (WPL)” which specifies the limit of PO_4_^3-^-P in wastewater discharges at the level not exceeding 0.8 mg L^−1^ (Preisner et al., 2020). Neither NSWW nor SWW had a post treatment concentration within the limit. Therefore, the need for additional treatment to remove PO_4_^3-^-P is crucial.

#### 3.3.4 Advantages and experimental limitations of organic matter and nutrient removal bioassays with MBC

The present study features four main limitations that are described as follows. (1) It was not possible to outline in detail the MBC growth nor nutrient removal since all experimental measurements were taken during the 12 h of light, that is when microalgae carried out photosynthetic activity, and no profiles during the other 12 h of darkness when no photosynthesis took place were evaluated. (2) NO_3_^−^-N concentrations were measured only at the beginning and end of the experiment. Hence, removal as a function of time cannot be appreciated. (3) The contribution of O_2_ generated by microalgae to nutrient removal dynamics *per se* could not be fully understood as the air was provided continuously throughout the experiment with a diffuser aeration of 1 L s^−1^ using an air pump. (4) It remains unknown which nutrients were eliminated by bacteria and which by microalgae. Therefore, it is highly recommended that in future experiments the following aspects should be considered: (1) perform nutrient measurements during dark period time, (2) determine NO_3_^−^-N concentrations more frequently, (3) permanently remove air supply and (4) include a dark control (absence of light).

The better performance of MBC in NSWW compared to SWW in terms of nutrient removal could be explained by the differences in media composition under non-sterilized (NSWW) and sterilized wastewater (SWW) conditions with the latter presenting lower initial concentrations of organic matter (total and soluble COD and BOD_5_) and nutrients (nitrogen and phosphorous). This can also be attributed to the presence of the autochthonous microbial community in the wastewater, which is probably boosting microalgal-bacterial interactions. Therefore, the suggested native MBC system was shown to be resilient with significant scalability potential, serving as a viable all-in-one solution for secondary and tertiary stages of wastewater treatment. This is particularly relevant in regions without wastewater treatment plants (WWTPs), a common scenario in many low-middle-income countries (LMIC). Future research should focus on the economic analysis of the microalgal-bacterial wastewater treatment system in terms of commercialization and up-scaling for potential applications (Khoo et al., 2021).

## 4 Conclusion

In this study, the capability of six native microalgal-bacterial consortia (MBC) from the Ecuadorian Amazon to efficiently treat domestic effluents in non-sterilized wastewater (NSWW) and sterilized wastewater (SWW) samples was comprehensively investigated. On average, COD and NH_4_^+^-N removal efficiencies in NSWW were higher than those in SWW by 9.53% and 23.90%, respectively. In fact, in NSWW, removal efficiencies reached up to 93.78 ± 0.57, 90.78 ± 3.14, 72.76% ± 8.97% and 53.46% ± 8.56% for COD, NH_4_^+^-N, PO_4_^3-^-P and NO_3_^−^-N, respectively. The enhanced performance of MBC in NSWW can be attributed to a potential synergy between the autochthonous microbial communities present in NSWW, but not in SWW. Media composition differences between NSWW and SWW, including reduced nutrient availability in SWW due to sterilization, could also have been attributed to observed differences in the bioremediation performance of native MBC in the two types of wastewater samples evaluated in this study.

The findings also underscore the influence of biodiversity when designing water remediation strategies based on MBC. Our results reveal differences in removal rates and efficiencies among the six native MBCs evaluated in this study, plausibly indicating that the diversity within each consortium and their respective origins play a crucial role in the removal capability of MBCs. Future research should focus on elucidating the taxonomic and functional profiles of microbial communities within the consortia, paving the way for a more comprehensive understanding of their potential applications in sustainable wastewater management.

## Data Availability

The original contributions presented in the study are included in the article/Supplementary Material, further inquiries can be directed to the corresponding author.
