# Supplementary material for: Native microalgal-bacterial consortia from the Ecuadorian Amazon region: an alternative to domestic wastewater treatment
Source: Front Bioeng Biotechnol. 2024 Feb 26;12:1338547. doi: 10.3389/fbioe.2024.1338547 (PMC10925762; doi:10.3389/fbioe.2024.1338547)
Supplement: Supplementary file 1 [file DataSheet1.pdf]

Supplementary Material

Native microalgal-bacterial consortia from the Ecuadorian Amazon Region: An alternative to domestic wastewater treatment

Amanda M. López-Patiño[1], Ana Cárdenas-Orrego[2], Andrés F. Torres[3], Danny Navarrete[1], Pascale Champagne[4], and Valeria Ochoa-Herrera[1,5,6 *]

[1] Colegio de Ciencias e Ingeniería, Universidad San Francisco de Quito USFQ, Diego de Robles y Vía Interoceánica, Quito 17-1200-841, Ecuador; alopez1@alumni.usfq.edu.ec (A.M.L.-P.); dnavarrete@usfq.edu.ec (D.N.); vochoa@usfq.edu.ec (V.O-H.)

[2] Instituto de Microbiología, Universidad San Francisco de Quito USFQ, Diego de Robles y Vía Interoceánica, Quito 17-1200-841, Ecuador; (A.-C.)

[3] Colegio de Ciencias Biológicas y Ambientales, Universidad San Francisco de Quito USFQ, Diego de Robles y Vía Interoceánica, Quito 17-1200-841, Ecuador; atorres@usfq.edu.ec (A.F.T.)

[4] Department of Civil Engineering, Queen's University, Kingston, K7L 3N6, Canada; pascale.champagne@queensu.ca (P.C.)

[5] Department of Environmental Sciences and Engineering, Gillings School of Global Public Health, University of North Carolina at Chapel Hill, Chapel Hill, NC 2759, USA

[6] Escuela de Ingeniería, Ciencia y Tecnología, Universidad del Rosario, Bogotá 111221, Colombia

* Correspondence: vochoa@usfq.edu.ec

Table S1. Initial (C_o) and final (C_f) concentrations of sCOD, NH_4^+-N, NO_3^--N and PO_4^{3-}-P of 6 native microalgal-bacterial consortia (MBC) from the Ecuadorian Amazon cultivated in NSWW and SWW.

WW Type	Bioassay	sCOD [mg L^{-1}]		NH_4^+-N [mg L^{-1}]		NO_3^--N [mg L^{-1}]		PO_4^{3-}-P [mg L^{-1}]	
		C_o	C_f	C_o	C_f	C_o	C_f	C_o	C_f
NSWW	AC	626.53	523.79	186.05	135.14	0.00	0.00	55.15	41.49
	T1	533.53	67.81	168.42	29.85	4.03	1.92	60.35	23.45
	T2	538.44	33.47	163.78	23.57	5.35	2.86	62.94	28.45
	T3	555.07	73.02	140.89	41.55	4.92	3.15	43.29	33.14
	T4	503.28	80.16	125.82	15.77	9.39	4.69	68.26	16.87
	T5	567.01	73.47	148.15	46.50	0.89	0.78	62.89	16.85
	T6	613.31	44.79	131.10	75.02	3.43	1.98	48.11	21.78
SWW	AC	591.94	458.96	82.85	75.41	2.30	2.01	41.61	33.30
	T1	716.94	112.42	82.85	27.15	1.88	1.69	44.50	9.20
	T2	578.06	51.92	70.91	33.00	11.51	2.46	39.30	6.50
	T3	591.94	145.53	64.74	31.68	6.60	2.23	42.18	5.80
	T4	633.61	62.68	79.21	34.07	4.59	1.46	39.30	0.00
	T5	655.83	55.12	81.91	37.46	2.13	1.01	50.85	6.07
	T6	594.72	68.48	73.49	43.56	3.32	2.09	39.30	0.00

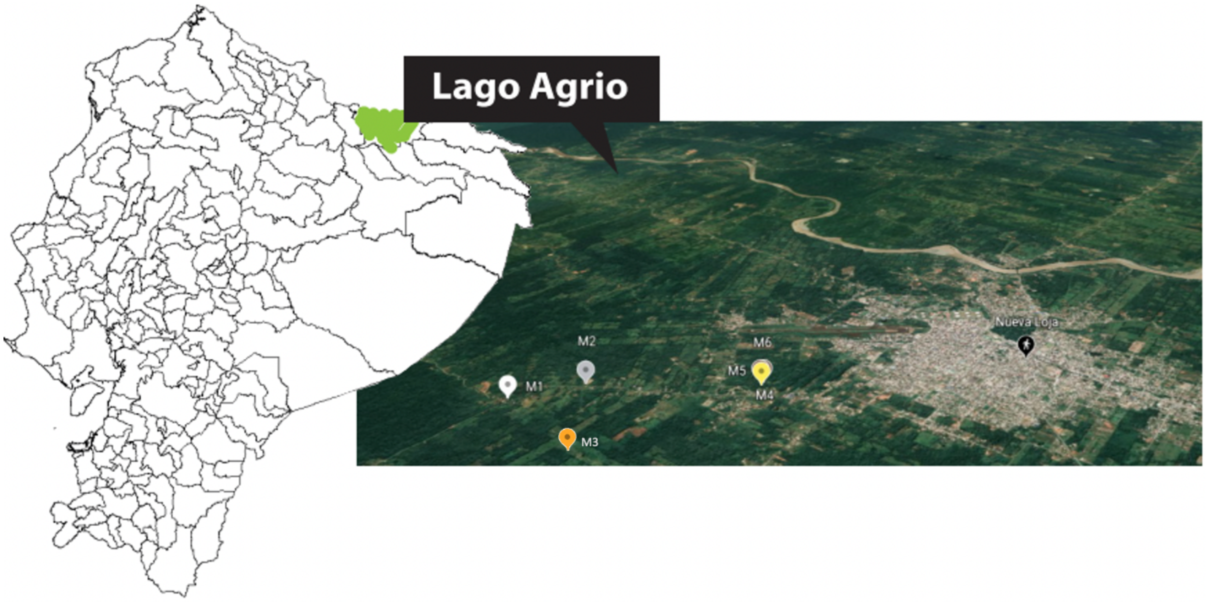

Supplementary Figure S1. Original geographical locations in Lago Agrio – Ecuadorian Amazon Region where native microalgal-bacterial consortia (MBC) were taken.
